# The Current State of Tinnitus Diagnosis and Treatment: a Multidisciplinary Expert Perspective

**DOI:** 10.1007/s10162-024-00960-3

**Published:** 2024-08-13

**Authors:** Tobias Kleinjung, Nicole Peter, Martin Schecklmann, Berthold Langguth

**Affiliations:** 1 Department of Otorhinolaryngology — Head and Neck Surgery, University Hospital Zurich, Zurich, Switzerland; 2 University of Zurich, Zurich, Switzerland; 3 Department of Psychiatry and Psychotherapy, University of Regensburg, Bezirksklinikum, Universitätsstr. 84, 93049 Regensburg, Germany; 4 Multidisciplinary Tinnitus Clinic, University of Regensburg, Regensburg, Germany

**Keywords:** Tinnitus, Evidence, Guidelines, Meta-analysis, Treatment

## Abstract

Tinnitus, the perception of sound without an external source, affects 15% of the population, with 2.4% experiencing significant distress. In this review, we summarize the current state of knowledge about tinnitus management with a particular focus on the translation into clinical practice. In the first section, we analyze shortcomings, knowledge gaps, and challenges in the field of tinnitus research. Then, we highlight the relevance of the diagnostic process to account for tinnitus heterogeneity and to identify all relevant aspects of the tinnitus in an individual patient, such as etiological aspects, pathophysiological mechanisms, factors that contribute most to suffering, and comorbidities. In the next section, we review available treatment options, including counselling, cognitive-behavioral therapy (CBT), hearing aids and cochlear implants for patients with a relevant hearing loss, sound generators, novel auditory stimulation approaches, tinnitus retraining therapy (TRT), pharmacological treatment, neurofeedback, brain stimulation, bimodal stimulation, Internet- and app-based digital approaches, and alternative treatment approaches. The evidence for the effectiveness of the various treatment interventions varies considerably. We also discuss differences in current respective guideline recommendations and close with a discussion of how current pathophysiological knowledge, latest scientific evidence, and patient perspectives can be translated in patient-centered care.

## Introduction

Tinnitus, which is characterized by the perception of sound without any external acoustic signal, affects approximately 15% of the population [1]. While the majority of individuals with tinnitus are not severely impaired, around 2.4% experience significant distress [1]. These individuals suffer from significant impairment and often have comorbidities such as hyperacusis, insomnia, anxiety, or depression, which adds to the complexity of their condition [2]. Recently, a new diagnostic classification has been proposed to distinguish between pure “tinnitus” and the more disabling “tinnitus disorder” [3].

In the field of tinnitus, a further distinction can be made between objective and subjective forms. Objective tinnitus, also known as “somatosound,” refers to internally generated sounds such as sounds caused by muscle movements or blood flow. This review focuses on chronic subjective tinnitus, where the perceived sound has no identifiable external or internal source. According to a recent consensus, article chronic tinnitus is defined by a duration of at least 3 months [3].

In this review, we summarize current state of knowledge about the diagnostic work-up and evidence-based treatment options for tinnitus, with a particular focus on their implementation into clinical practice. With authors representing various disciplines (otology and neurotology (T. K.; N. P.), psychology (M. S.), psychotherapy (M. S.: B.L), and neurology and psychiatry (B.L.)), we want to emphasize the need for a multidisciplinary approach for tinnitus management.

## Shortcomings and Challenges in Tinnitus Management

The diagnostic and therapeutic landscape for tinnitus patients varies considerably across countries and even within regions. Patients may receive different treatments depending on which institution they approach, reflecting the current lack of standardized, evidence-based care [4]. There are also differences in the assessment of individuals with tinnitus, influenced by factors such as point of contact, cultural differences, and variations in the healthcare system. Ideally, an evidence-based, stepwise diagnostic approach should be implemented universally, regardless of the first point of contact within the healthcare system [5]. Similarly, treatment decisions should be tailored to the individual patient rather than the treating clinician’s specialty. Both patients and clinicians are dissatisfied with the current state of tinnitus management [4]. Given the prevalence, significant impact on affected individuals, and chronic nature of tinnitus, there is an urgent need for effective treatments.

The current unsatisfactory state of tinnitus management [4] is due to several factors. First, there is no treatment that can reliably eliminate tinnitus or reduce its loudness, which would be the main wish of most patients [6–9]. Although there are reports of single cases of tinnitus remission, no causal relationship has been established between specific therapeutic interventions and the disappearance of tinnitus in these patients [10]. However, there are many evidence-based options to reduce tinnitus suffering [4].

Tinnitus exhibits significant heterogeneity in terms of clinical features, pathophysiology, and response to treatment [11–13]. Efforts to classify different clinical subtypes have had limited success [14–16]. Profiling patients according to dimensions such as tinnitus severity, somatosensory impact, and hearing loss may be more pragmatic [16, 17]. Furthermore, the current understanding of the pathophysiology of the different forms of tinnitus is still incomplete [18], impeding progress in the development of effective treatments. Imaging, electrophysiologic, and genetic studies provide mixed results, and the limited power of animal models makes matters even more challenging [18]. Unlike serendipitous discoveries in other medical fields, such breakthroughs in tinnitus research have so far failed to occur [19].

The lack of established biomarkers and objective outcome measures complicates clinical research. Standardized questionnaires have been developed to assess various aspects of tinnitus, including distress, handicap, and functional impairment [20]. A longer-lasting reduction in tinnitus loudness, which is desired by most patients [6], can currently only be achieved by cochlear implants for unilateral deaf patients [21]. Reductions in tinnitus loudness due to phenomena such as residual inhibition are only short term and not permanent. Tinnitus loudness can be assessed by psychophysical methods such as loudness matching or minimal masking level or by subjective assessment using scales such as visual analogue or numeric rating scales. However, psychophysical measurements have a low reliability, and do not reflect subjective changes. As the subjective assessment of loudness can be affected by distress, the determination of tinnitus loudness remains complicated [22].

In clinical trials, therefore, the gold standard for primary outcome measurement is the use of tinnitus questionnaires, which provide a more comprehensive and reliable assessment of the multiple manifestations of tinnitus [23, 24]. In contrast, measures of tinnitus loudness, although valuable, are of secondary importance as outcome measures in such trials. The clinical relevance of score reductions of existing questionnaires [25] and whether they adequately cover all relevant domains of tinnitus impairment [26] are the subject of ongoing debate.

## Comprehensive Diagnostic Assessment of Tinnitus as a Basis for Treatment

Usually, recommendations for diagnostic assessment by means of history taking and clinical examination are based less on evidence from systematic controlled trials but rather on expert recommendations. This is also the case in the tinnitus field. Here, we summarize recommendations from guidelines [27] [28] and the authors’ clinical experience [29, 30].

At the beginning of each assessment, it is crucial to realize that tinnitus can create a high degree of uncertainty and psychological stress. Patients may fear that tinnitus may indicate a serious underlying condition, even though such cases represent only a small minority. The initial case history interview, therefore, requires a high degree of seriousness, reliability, and empathy. It is equally important to conduct this conversation carefully in order to avoid unnecessary sensitization and to prevent an excessive focus on the symptom and the development of unnecessary fears. An essential goal is to live with chronic tinnitus without handicap, which means with a minimum of emotional and physiological stress and without avoiding activities, which improve quality of life. It is important to find a balance between awareness for tinnitus conditions at the beginning of the diagnostic process (e.g., to find out relevant causal and modulating factors) and the long-term strategy of avoiding sensitization to the symptom, as it is the therapeutic idea in psychosomatic disorders.

Subsequently, it is important to ask about the circumstances that led to the onset of tinnitus, such as noise exposure, ear infections, or stressful situations. The duration of the symptoms and the examination of accompanying otological symptoms such as perceived hearing loss, dizziness, or otalgia are also important parts of the diagnostic process. The laterality and quality of the sound are of critical importance, as unilateral sounds may indicate a structural origin, which can potentially be treated causally. Determining whether the sound is continuous, rhythmically pulsatile, or pulse synchronous provides valuable information about the nature of the tinnitus, with pulsatile symptoms warranting imaging, particularly if they persist for more than 3 months.

Pure-tone audiometry should be performed in every tinnitus patient, even if no impairment is perceived, as it can reveal subtle hearing loss that may impact speech comprehension or occur in ultrahigh-frequency ranges [31]. In addition to its diagnostic value, pure-tone audiometry provides guidance for potential therapeutic interventions such as hearing aids and informing discussions about the conceptual development of tinnitus. For non-pulsatile unilateral tinnitus, imaging, such as MRI of the temporal bone, is recommended, especially if an asymmetrical hearing loss is ipsilateral to the tinnitus side.

Active exploration of possible psychological distress related to tinnitus and previous distressing circumstances is crucial. By supplementing interviews with standardized questionnaires, such as Tinnitus Handicap Inventory (THI) [32] and Tinnitus Functional Index (TFI) [33], the severity of distress can be quantified, providing clinicians with guidance towards psychiatric/psychotherapeutic assessment and treatment.

Active questioning for possible modulation of tinnitus through manipulation of the neck and masticatory muscles is recommended. Such modulation suggests a somatic tinnitus component and opens up possibilities for physiotherapeutic interventions [34, 35].

Finally, it is important to identify the primary source of distress related to the patient’s tinnitus, as this serves as an important guide for treatment. For instance, if a patient’s primary complaint is tinnitus-induced insomnia, the treatment plan should incorporate strategies to address and alleviate insomnia. The question about the primary source of distress should be complemented by exploration of comorbidities such as anxiety, depression, insomnia, hyperacusis, communication difficulties, temporomandibular joint disorder, headache, or neck pain. These comorbidities can have a negative impact on tinnitus and also increase the overall burden of the patients. Therefore, the treatment plan should also address these comorbidities.

In summary, a comprehensive diagnostic work-up should explore underlying mechanisms and comorbidities of tinnitus in auditory, somatosensory, and psychological domains. Establishing an understanding of atmosphere is essential for fostering a therapeutic relationship. Based on the diagnosis, informative counselling sessions can be initiated, and potential somatic therapy approaches can be evaluated. For persistent and severely impairing tinnitus symptoms, a specialized interdisciplinary team provides an ideal setting for a comprehensive assessment and treatment to improve overall quality of life.

## Treatment of Tinnitus

Many different tinnitus treatments have been proposed. Box 1 provides a (possibly incomplete) overview of therapeutic interventions, which have been investigated in clinical trials. Due to the limited knowledge of the pathophysiological mechanisms of tinnitus, most approaches have only a weak pathophysiological rationale and follow a “trial-and-error” approach.

Box 1 Therapeutic interventions for tinnitus that have been evaluated with randomized controlled trials (listed in alphabetical order; treatments recommended by most guidelines are listed in bold and marked with “recommended by guidelines”; see also Table 1, modified from [36]).

**Table 1:** Evidence for several tinnitus treatments and recommendations of the various guidelines (listed in alphabetical order; modified from [36])

Intervention	Source of evidence	Number of study participants	Efficacy (immediate)	Efficacy (long term)	Potential harm	US (2014)	European (2019)	NICE (2020)	German (2021)
Anticonvulsants	Cochrane [59]	453	Insufficient evidence	Not reported	Side effects reported in 18% of participants	Clinicians should not routinely recommend anticonvulsants for a primary indication of treating persistent, bothersome tinnitus (recommendation against)	Weak recommendation against pharmacological treatment	Not mentioned	Strong recommendation against pharmacological treatment
Antidepressants	Cochrane [60]	610	Insufficient evidence	Not reported	Side effects common	Clinicians should not routinely recommend antidepressants for a primary indication of treating persistent, bothersome tinnitus (recommendation against)	Weak recommendation against pharmacological treatment	Not mentioned	Strong recommendation against pharmacological treatment
Auditory training	Systematic review [48]	269	Available evidence of insufficient quality to make conclusion about efficacy	Not reported	Not reported	Not mentioned	Not mentioned	Not mentioned	Recommendation for auditory training
Betahistine	Cochrane [74]	303	No significant effects on tinnitus loudness or distress	Not reported	Side effects on placebo level	Not mentioned	Weak recommendation against pharmacological treatment	Do not offer betahistine to treat tinnitus	Strong recommendation against pharmacological treatment
Cochlear implant	Meta-analysis [75]	674	Tinnitus score (SMD: − 1.32)	Not reported	not reported	Not mentioned	No recommendation for cochlear implants	Not mentioned	Strong recommendation for cochlear implants in patients with tinnitus and severe hearing loss/deafness
Cognitive behavioral therapy	Cochrane [42]	2733	Tinnitus severity (SMD): − 0.56 THI: − 10.91	No evidence due to lack of data	Adverse effects are rare	Clinicians should recommend CBT to patients with persistent, bothersome tinnitus (recommendation)	Strong recommendation for cognitive behavioral therapy	If tinnitus is still causing an impact on emotional and social wellbeing, and day-to-day activities, consider a stepped approach as follows: 1. Digital tinnitus-related cognitive behavioral therapy (CBT) 2. Group-based tinnitus-related psychological interventions including mindfulness-based cognitive therapy, acceptance, and commitment therapy or CBT 3. Individual tinnitus-related CBT	Strong recommendation for cognitive behavioral therapy
Dexamethasone (intratympanic administration)	Meta-analysis [76]	220	No significant effect compared with placebo	No significant effect compared with placebo	Complications such as hearing loss, eardrum perforation, and middle ear inflammation are rare	Clinicians should not routinely recommend intratympanic medications for a primary indication of treating persistent, bothersome tinnitus (recommendation against)	Weak recommendation against pharmacological treatment	Not mentioned	Strong recommendation against pharmacological treatment
Ginkgo biloba	Cochrane [58]	1915	Little to no effect at 3 to 6 months compared to placebo, but the evidence is very uncertain	Little to no effect at 3 to 6 months compared to placebo, but the evidence is very uncertain	Incidence of side effects, low	Clinicians should not recommend Ginkgo biloba, for treating patients with persistent, bothersome tinnitus (recommendation against)	Weak recommendation against pharmacological treatment	Not mentioned	Strong recommendation against pharmacological treatment
Hearing aid	Cochrane [47]	590	No significant effects on tinnitus loudness or distress	No data	Not reported	Clinicians should recommend a hearing aid evaluation for patients with hearing loss and persistent, bothersome tinnitus (recommendation)	Weak recommendation for hearing aids	Offer amplification devices to people with tinnitus who have a hearing loss that affects their ability to communicate Consider amplification devices for people with tinnitus who have a hearing loss but do not have difficulties communicating Do not offer amplification devices to people with tinnitus but no hearing loss	Recommendation for hearing aids in case of hearing loss
Hyperbaric oxygen	Cochrane [77, 78]	392	No significant improvements in tinnitus for chronic tinnitus	No significant improvements in tinnitus for chronic tinnitus	Not reported	Not mentioned	Not mentioned	Not mentioned	Not mentioned
Sound therapy	Cochrane [47]	590	No significant effects on tinnitus loudness or distress	No data	Not reported	Clinicians may recommend sound therapy to patients with persistent, bothersome tinnitus. (option)	No recommendation	Recommendation for research	Recommendation against sound generators, recommendation against specific sound therapies
Tinnitus retraining therapy	Meta-analysis [56]	1345	Significantly increased treatment response	Significantly increased treatment response	Not reported	Not mentioned	No recommendation	Not mentioned	Can be considered for long-term treatment
Transcranial direct current stimulation	Meta-analysis [79]	1031	Loudness (SMD) − 0.35 Distress (SMD): − 0.5	Not reported	Not reported	Not mentioned	No recommendation for transcranial electrical stimulation	Recommendation for research	Recommendation against transcranial electrical stimulation
Transcranial magnetic stimulation	Meta-analysis [63, 80]	945	Tinnitus severity (SMD): − 0.45	Tinnitus severity (SMD): − 0.42	Not reported	Clinicians should not recommend TMS for the routine treatment of patients with persistent, bothersome tinnitus (recommendation against)	Recommendation against transcranial magnetic stimulation	Recommendation for research	Recommendation against transcranial magnetic stimulation
Zinc	Cochrane [81]	209	No evidence for improvement of tinnitus severity by oral zinc supplementation	No evidence for improvement of tinnitus severity by oral zinc supplementation	Not reported	Clinicians should not recommend zinc, for treating patients with persistent, bothersome tinnitus (recommendation against)	Weak recommendation against pharmacological treatment	Not mentioned	Strong recommendation against pharmacological treatment

**Table:** 

**Pharmacological interventions**
• *Antidepressants*
Amitriptyline
Nortriptyline
Paroxetine
Sertraline
Trimipramine
• *Anticonvulsants*
Carbamazepine
Gabapentin
Lamotrigine
Selurampanel
• *Benzodiazepines/GABAergic drugs*
Alprazolam
Baclofen
Clonazepam
Diazepam
• *Glutamatergic drugs*
Acamprosate
Esketamine
Memantine
Neramexane
• *Muscle relaxants*
Cyclobenzaprine
Eperisone
Orphenadrine
Tizanidine
• *Sodium channel blocker*
Lidocaine
• *Others*
Atorvastatin
Betahistine
Chinese medicine
Cilostazol
Cyclandelate
Deanxit
Ginkgo biloba
Melatonin
Misoprostol
3,4-Methylenedioxymethamphetamine (MDMA)
Naloxone
Ondansetron
Oxytocin
Piribedil
Pramipexole
Vardenafil
Vitamin B12
Zinc
**Non-pharmacological interventions**
• *Acupuncture/acupressure*
• *Bimodal stimulation*
Electrical vagus nerve stimulation plus sound therapy
Electrical skin stimulation plus sound therapy
Electrical tongue stimulation plus sound therapy
• *Brain/neural stimulation*
Transcranial magnetic stimulation
Transcranial direct current stimulation
Direct electrical stimulation
Vagus nerve stimulation
Transcutaneous electrical neural stimulation
• *Combination approaches*
Tinnitus retraining therapy (directive counselling plus sound therapy)
Neuromonics (counselling plus sound therapy)
• *Electrical stimulation of the ear/cochlea*
**Cochlear implants**
(“Recommended by guidelines” in case of profound hearing loss)
Electrical stimulation of the tympanum or the outer ear canal
• ***Hearing aids***
(“Recommended by guidelines” in case of hearing loss)
• *Hyperbaric oxygenation*
• *Low-level laser therapy*
• *Music therapy*
• *Neurobiofeedback*
• *Physiotherapy*
• *Psychotherapy*
**Cognitive behavioral therapy (individual, group, or online setting)** (“recommended by guidelines”)
**Counselling/psychoeducation** (“recommended by guidelines”)
Mindfulness-based therapy
Hypnosis
• *Sound therapy*
Noise generator (complete masking)
Noise generator (partial masking)
Enriched acoustic environment
Fractal tones
Auditory training
Tailor-made notched music training
Coordinated reset auditory stimulation
• *Virtual reality-based approaches*

### Tinnitus Counselling

Tinnitus counselling is a therapeutic process in which individuals with tinnitus are supported and guided by a trained professional to help them cope with the psychological and emotional aspects of their condition. Counselling aims to reduce the impact of tinnitus on the individual’s quality of life by providing information about tinnitus, discussing coping strategies, exploring stress management techniques, and offering relaxation exercises, emotional support, and self-efficacy enhancement [30, 37]. Tinnitus counselling is considered a fundamental therapeutic approach recommended by all guidelines, although evidence from randomized controlled trials is limited [36, 38]. Recent studies exploring counselling via smartphone apps [39] promise wider accessibility and increased patient involvement.

### Cognitive Behavioral Therapy (CBT) for Tinnitus

Cognitive behavioral therapy (CBT) for tinnitus is a treatment that incorporates cognitive, behavioral, or a combination of components in a structured time-limited program, aiming to change the negative thought patterns and behaviors associated with tinnitus [40]. CBT typically involves identifying and challenging negative thoughts related to tinnitus, developing relaxation techniques, and habituation to the tinnitus sound by distraction techniques. Another approach for cognitive restructuring is the systematic exposition to the tinnitus sound through a mechanism similar to the treatment of phobias. The goal of CBT is to help patients better control their emotional reactions and improve their ability to cope with the impact of tinnitus on their daily lives [41]. CBT for tinnitus treatment has been investigated in a large number of clinical trials [42]. The results of a Cochrane meta-analysis suggest that tinnitus questionnaire scores are effectively reduced after CBT treatment with minimal adverse effects. Both face-to-face and Internet-delivered CBT demonstrate comparable efficacy [42]. As there are various behavior modification techniques, it would be desirable to identify predictors to determine the best CBT technique for the individual patient. For example, exposure as a treatment technique is supposed to have best effects in patients who fear their tinnitus and therefore avoid silent environments [43]. Mindfulness-based interventions, considered as part of CBT, are a promising way to reduce tinnitus distress, as recent systematic reviews indicate [44]. However, the long-term effects are still uncertain, and further research is needed to determine their effectiveness.

### Auditory Treatments

Auditory treatments include a wide range of interventions, such as devices for hearing improvement, sound generators for masking or for distracting from tinnitus, and various auditory stimulation techniques. In cases of profound hearing loss, cochlear implants have demonstrated a significant reduction in tinnitus perception and distress [21, 45]. Consequently, unilateral burdensome tinnitus in single-sided deafness may indicate the need for cochlear implantation, even if the ability to communicate is preserved in most acoustic environments by the normal contralateral hearing function. Hearing aids are recommended when there is an indication due to comorbid hearing loss [36]. However, the use of hearing aids as a tinnitus treatment in patients with adequately preserved communication skills, e.g., mild hearing loss or hearing loss in the high-frequency range, is controversial due to the low quality of evidence [46, 47]. Similarly, there is insufficient evidence supporting the effectiveness of sound generators or other forms of sound therapy, although clear benefits have been observed in a subset of patients [47]. Music therapy and hearing trainings have been proposed as an option for the treatment of tinnitus with or without hearing loss, but the evidence base for their effectiveness is limited [37, 48].

Various individualized auditory stimulation approaches tailored to an individual’s tinnitus frequency or hearing profile have been proposed [49–51]. These approaches aim to induce specific neuroplastic changes and have shown promising pilot data, but the effects have not yet been validated in independent larger controlled studies. Either such studies are still missing or they revealed negative results [52–54].

In general, many patients seem to benefit from acoustic distraction, be it music, white noise, or natural sounds, in certain situations such as falling asleep or concentrating.

### Tinnitus Retraining Therapy (TRT)

Tinnitus retraining therapy (TRT) is a combination of directive counselling and sound therapy, either through hearing aids or noise generators, aiming at habituation [55]. Despite its widespread clinical use, evidence for its efficacy is limited [56]. In a large randomized controlled trial, TRT and its components were compared with standard of care treatment. Patients were randomly assigned to either TRT (counselling + sound therapy), partial TRT (only counselling), or standard treatment with no significant differences found between groups [57]. A recent meta-analysis indicated a potential positive long-term effect of TRT, but further well-designed studies are needed to clearly demonstrate its effectiveness [56].

### Tinnitus Pharmacotherapy

Despite numerous studies, pharmacotherapy for tinnitus has produced predominantly null results in meta-analyses [58–60]. Accordingly, there is no drug that has been approved by the Food and Drug Administration (FDA) or the European Medical Agency (EMA) for the treatment of tinnitus [61, 62]. Current guidelines recommend pharmacological treatment only for the therapy of comorbidities such as insomnia, depression, or anxiety [36].

### Neurobiofeedback and Brain Stimulation

Neurobiofeedback is promising based on controlled studies, but more extensive confirmatory research is needed. Noninvasive brain stimulation, including transcranial direct current stimulation (tDCS) and repetitive transcranial magnetic stimulation (rTMS), demonstrates positive effects with small to moderate effect sizes and offers potential therapeutic opportunities [63]. Currently, it is not recommended by any guideline, also due to the fact that most studies are rather recent, and that the required level of evidence for innovative treatments to be included in guidelines is particularly high [36, 64]. Invasive brain stimulation remains highly experimental, with beneficial outcomes reported in case series but insufficient data to support routine clinical use [65].

### Bimodal Stimulation

Bimodal stimulation approaches, in which auditory stimuli are combined with various forms of neural stimulation, are promising for reducing tinnitus severity. Auditory stimulation combined with electrical stimulation of the tongue has shown beneficial effects in first studies in large samples [66, 67]. The combination of auditory stimulation with electrical face or neck stimulation [68] or vagus nerve stimulation [69] indicates substantial benefits in pilot studies, highlighting the need for further investigation.

### Complementary and Alternative Therapies

Among complementary therapies, acupuncture is one of the best studied but has shown mixed results [70]. Many other techniques have been proposed and tested (see also Box 1), mainly in small uncontrolled trials. The evidence is inconclusive and requires more rigorous research.

### Physiotherapy and Manual Therapy

Based on the knowledge about somatosensory influences on tinnitus [71], physiotherapy and manual therapy have been investigated in patients with somatosensory tinnitus [35], with promising results [34], and further systematic studies are warranted [72].

### Self-Help Interventions

Traditional self-help interventions, facilitated through mutual self-help groups, emphasize social integration and psychosocial relief. However, clear evidence regarding their efficacy for tinnitus remains elusive. The term “self-help” has also been applied to online CBT and other smartphone-based interventions where the potential for tinnitus management is currently being explored [73]. The concepts of self-help should be defined in detail for the research perspective. First, a clear distinction should be made between online-guided treatments and mutual support for patients in self-help groups. Secondly, a differentiation should be established with regard to the type, nature, and content of self-help activities.

## Overview of Treatments, Their Results, and Guideline Recommendations

Table 1 provides an overview of the best-studied tinnitus treatments, their evidence, and the respective recommendations in current guidelines.

In summary, treatment options for tinnitus are diverse and range from traditional counselling to innovative technology-based interventions. Whereas some modalities show promise, further in-depth research is needed to establish robust evidence-based guidelines for more effective tinnitus management.

## Advancing Tinnitus Management

### Inconsistencies in Tinnitus Management

Tinnitus management varies widely around the world, reflecting differences between countries, medical disciplines, and healthcare institutions [4, 82]. Given this diversity, the development of treatment guidelines based on current evidence represents a crucial step towards establishing a standardized approach [5, 36, 38, 83–85]. The evidence-based treatment options currently available are limited, highlighting the urgent need for improved and innovative solutions.

### Challenges in Guideline Development

Guidelines tend to be conservative by nature and focus on established treatments based on experience, eminence, and evidence. However, this should not discourage the exploration of innovative approaches [36]. Overcoming this dilemma requires strategic considerations. Practically, all meta-analyses in the field of tinnitus emphasize the need for larger studies with higher methodological rigor. In addition, clinical trials should take into account the inherent heterogeneity of tinnitus. There is always a certain risk of bias in guideline committees, as most experts have material or immaterial conflicts of interest. One solution would be for the evidence to be assessed by independent guideline committees with methodological expertise, as is the case with the NICE guidelines. It is possible that AI applications might be developed in the near future to facilitate this time-consuming work. The methodology of guideline development contains a certain bias towards established treatments. Therefore, care should be taken to ensure that that guidelines remain open to novel therapeutic options. For example, rather than advising against innovative interventions due to insufficient safety or efficacy data, guidelines should emphasize the need for research. A good example of this are the NICE guidelines, which make explicit recommendations on research priorities based on the lack of evidence for the aspects of management they review. Faster update cycles should also be introduced. Adherence to the concept of a “living guideline” allows timely incorporation of new evidence and improves adaptability to emerging treatments. Finally, it is important to actively involve tinnitus patients in guideline development to ensure that their perspective contributes to a comprehensive understanding of the condition and its treatment [86].

### Development of Decision Support Systems

The current treatment landscape for tinnitus is characterized by a variety of options, each with varying outcomes. While some patients experience significant improvement from a particular treatment, others achieve no benefit. This variability is not surprising given the diverse nature of tinnitus. However, predicting response to treatment based on specific clinical or demographic factors remains challenging. Consequently, patients often go through a trial-and-error process, attempting multiple treatments until they experience relief [87]. This approach is both burdensome and costly. There is hope that the situation can be ameliorated in future through the development of decision support systems. These systems, which use artificial intelligence to analyze large databases, aim to predict treatment response by taking into account a combination of various individual characteristics [88, 89].

### Balancing Evidence-Based Practice and Therapeutic Freedom

It is crucial to recognize that guidelines are not rigid laws but rather a dynamic framework based on the available evidence. In the clinical situation, guideline recommendations have to be translated into treatment options for the individual patient. Clinicians should keep in mind that evidence for diagnostic or therapeutic interventions should not be considered as categorical (“yes” or “no”) but rather as dimensional (“more” or “less”). In order to make informed treatment decisions, clinicians need to balance the existing evidence and guideline recommendations with individual factors and patient’s preferences. Clinicians’ therapeutic freedom also includes the option to offer treatments that are not or not yet recommended by guidelines. However, this requires good reasons, either new evidence for innovative treatments that has not yet been included in guidelines [67] or pathophysiological considerations, e.g., offering migraine medication for episodic tinnitus, which is similar to a symptom of cochlear migraine [90] or carbamazepine for “typewriter” tinnitus [91, 92]. For all therapeutic recommendations, but particularly for off-label treatments, the chances of improvement, treatment risks, and alternative options must be weighted and discussed with the patient.

### Striving for Quality Improvement

In conclusion, following these strategies can elevate the quality standards of tinnitus management through evidence-based guidelines while creating an environment conducive to innovation. The ultimate goal remains patient-centered care, where guidelines serve as valuable tools rather than rigid directives. By integrating the latest evidence, patient perspectives, and innovative approaches, the healthcare community can collaboratively advance the field of tinnitus management and improve outcomes for those affected by this difficult condition.
